# Melatonin blood values and total antioxidant capacity in critically ill patients

**DOI:** 10.1186/cc13626

**Published:** 2014-03-17

**Authors:** LA D'Amato, G Mistraletti, D Longhi, IR Piva, F Marrazzo, C Villa, M Tozzi, R Paroni, E Finati, G lapichino

**Affiliations:** 1 Università degli Studi di Milano, Milan, Italy

## Introduction

Endogenous melatonin is decreased in critically ill patients; oral supplementation could help them to cope with sleep disruption and sepsis. Among patients who took part in a trial on melatonin and need for sedation [1], some were studied with blood sampling to describe their melatonin blood values and the relationship with total antioxidant capacity (TAC) in plasma.

## Methods

Inclusion criteria: mechanical ventilation previewed at ICU admission >8 days and mortality predicted at ICU admission over 13% (SAPS II >32 points). After the clinical run-in period of 48 hours, samplings were taken to measure baseline blood melatonin and TAC, beginning from the third ICU night and day (midnight and 02:00 p.m.). At 8:00 p.m. of the third ICU day, treatment with 3 mg + 3 mg melatonin (Group M) or placebo (Group P) began: each patient received two tablets per day, at 8:00 p.m. and midnight, until ICU discharge. Further samplings were taken during the early (fourth night and day) and the late (eighth night and day) treatment phases. Melatonin was measured through an ELISA essay; TAC was measured with a specific kit.

## Results

Sixty-four critically ill patients were enrolled. Endogenous melatonin was shown decreased in the run-in period and in the placebo group compared with healthy subjects. All patients reached satisfying pharmacological values with enteral administration: peak of blood melatonin value (pg/ml) was 2,514 (982 to 7,148) for the M group versus 20 (15 to 62) for the P group (*P *< 0.001) during the first treatment night, while maintaining significant differences also during the daytime: 51 (23 to 180) M group versus 14 (11 to 24) P group (*P *= 0.001). The same trend was observed in the late treatment samples (eighth ICU day). Regarding TAC values (nmol Trolox equivalent/μ! plasma), a significant difference was highlighted during the night (107 (97 to 123) M group vs. 61 (42 to 89) P group, *P *< 0.001), but not during the daytime (37 (30 to 69) M group vs. 28 (25 to 50) P group, *P *= 0.092). Correlation between melatonin and TAC: Spearman's rho = 0.33 (*P *< 0.001).

## Conclusion

Enteral administration of melatonin was adequate in the early phase of critically illness, with pharmacokinetics similar to published data [2]. The administration of melatonin seems to increase the TAC, with a possible meaningful role in critically ill patients.
